# Polymer-Based Nanocarriers for Co-Delivery and Combination of Diverse Therapies against Cancers

**DOI:** 10.3390/nano8020085

**Published:** 2018-02-03

**Authors:** Guowen Yan, Aihua Li, Aitang Zhang, Yong Sun, Jingquan Liu

**Affiliations:** 1 School of Materials Science and Engineering, Qingdao University, No. 308 Ningxia Road, Qingdao 266071, China; yanguowen@qdu.edu.cn (G.Y.); aihuali@qdu.edu.cn (A.L.); 2015020658@qdu.edu.cn (A.Z.); 2 School of Pharmacy, Qingdao University, No. 38 Dengzhou Road, Qingdao 266021, China; sunyong@qdu.edu.cn

**Keywords:** nanocarrier, polymer, chemotherapy, photothermal therapy, cancer, combined therapy

## Abstract

Cancer gives rise to an enormous number of deaths worldwide nowadays. Therefore, it is in urgent need to develop new therapies, among which combined therapies including photothermal therapy (PTT) and chemotherapy (CHT) using polymer-based nanocarriers have attracted enormous interest due to the significantly enhanced efficacy and great progress has been made so far. The preparation of such nanocarriers is a comprehensive task involving the cooperation of nanomaterial science and biomedicine science. In this review, we try to introduce and analyze the structure, preparation and synergistic therapeutic effect of various polymer-based nanocarriers composed of anti-tumor drugs, nano-sized photothermal materials and other possible parts. Our effort may bring benefit to future exploration and potential applications of similar nanocarriers.

## 1. Introduction

Cancer, a torturing disease that threats human health, accounts for an enormous number of deaths worldwide nowadays, constituting a huge burden on the societies of both more and less developed countries [1]. Worse still, the relapse of drug-resistant tumors may allow neoplasm regeneration in the same place while the metastases of tumors allow dissemination to other places, which may diminish or even cripple anti-cancer treatments, leading to ascending mortality and insufficient prognosis [2,3,4,5,6,7,8,9]. Drug-resistance may develop as tumors adapt to and fight against the toxicity of drug [10]. Metastasis is the inherent property of some tumors [11]. Consequently, it is a major clinical challenge to evade potential relapse of drug-resistant cancer, e.g., the notorious drug-resistant kidney cancer that is often caught only after metastasis [12,13].

Although conventional chemotherapy (CHT) performed with small-molecule drugs plays a key role in the combat against cancer, it still shows some limitations, e.g., severe side effects, lack of selectivity (drugs enter healthy cells or tissues, leading to undesirable systematic toxicity and low therapeutic efficacy to tumors), poor water-solubility (hampering distribution in bloodstream and leading to low bioavailability) and multidrug resistance of tumor cells (decreasing therapeutic efficacy) [14,15,16,17,18,19].

With the ability to avoid the above limitations, polymer-based nanocarriers, which are nanoparticles capable of loading and delivering small-molecule agents, have gained increasing interest and became a focus of research [20]. However, previous nanocarriers generally load one drug only [21], which may be less lethal to tumor cells, especially multi-drug resistance (MDR) tumor cells [22,23]. It is possibly difficult for these single-drug nanocarriers to inhibit recurrence or metastasis of tumors.

Photothermal therapy (PTT), a treatment that external near-infrared (NIR) light is converted to heat by photoabsorbers (photothermal materials that emit heat following absorb light) to destruct adjacent cells or tissues by increasing the temperature, has attracted increasing attention in anti-cancer research [24,25]. However, owing to the limited radiant power of NIR, unsatisfied penetration depth and inhomogeneous heat distribution in tissues, PTT per se is not always able to completely remove tumors [26,27].

In consideration of the strengths and weaknesses of the both therapies above, PTT and CHT can be complementary to each other [28]. Namely, the combination of them can be more competent. Recently, such combined therapies for synergistic effect (the effect of two or more modalities is more than the sum of their individual effects) on tumors have emerged [29,30,31]. According to some reports, the synergistic of CHT plus PTT may eradicate in vivo tumors, largely preventing recurrence or metastasis [19,32]. We found that the common factor of the above successful combinational therapy is nanocarriers.

**Scheme 1:**
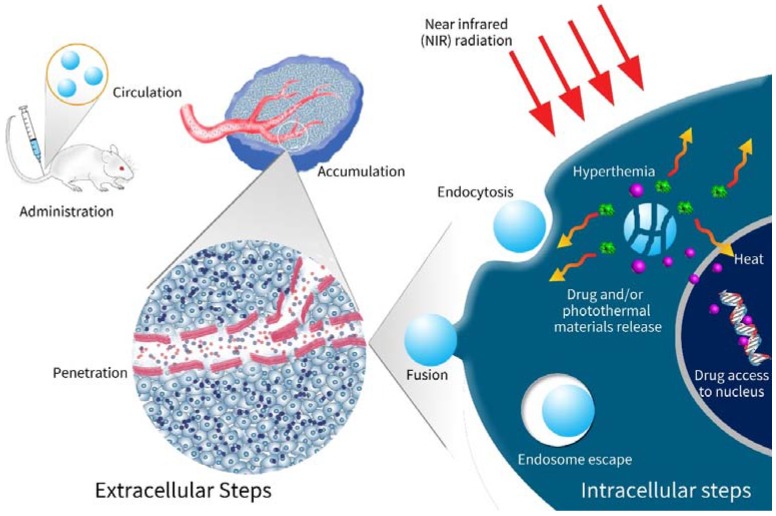
Schematic illustration of nanocarriers exerting therapeutic effects in vivo. After administration, the drug-loaded photothermal nanocarriers navigate to tumors through the blood circulation. Owing to the enhanced permeability and retention (EPR) effects, the nanocarriers penetrate tumor tissues across vessel walls of tumor vasculature, leading to accumulation within tumors. The nanocarriers then enter tumor cells via endocytosis. If targeting ligands exist, the endocytosis may be enhanced. After entrance into cells, the nanocarriers release drugs and emit heat under NIR radiation, leading to synergistic effects of CHT and PTT.

The integration and co-delivery of both drugs and photoabsorbers with double-cargo nanocarriers are feasible: (1) nanocarriers have plenty space to accommodate both agents [30,33,34]; (2) some nanoscale photothermal materials per se can load drugs to constitute nanocarriers [29,35,36]; (3) photoabsorbers produce heat that can trigger the release of drugs [14,37]; (4) the hydrophilic moiety of the polymer improves the bioavailability, stability and security of nanocarriers [9]. In summary, the in vivo route and action of the double-cargo nanocarriers are illustrated in Scheme 1.

Fabricating nanocarriers for combination of CHT and PTT belongs to the intersection of biomedicine and material science. Having retrieved articles published since 2016 from ISI Web of Knowledge and NCBI PubMed, we herein give a comprehensive overview on structure, preparation and synergistic therapeutic effect of various polymer-based nanocarriers composed of anti-tumor drugs, nano-sized photothermal materials and other possible parts. We try to seek potential rules over the fabrication and application via classification and induction of them. Finally, our effort may bring benefit to future exploration and potential application of similar nanocarriers.

## 2. The Basics of Polymer-Based Nanocarriers

Avoiding the limitations of small-molecule drugs, polymer-based nanocarriers that load and deliver anti-tumor agents have gained increasing interest and became a focus of research [20]. The polymer that composes nanocarriers are frequently amphiphilic, that is, have a hydrophobic moiety and a hydrophilic one. Via the hydrophobic moiety, nanocarriers may bind a multitude of drug molecules [38]. Due to the hydrophilic moiety such as polyethylene glycol (PEG) or chitosan (CTS), nanocarriers with several hundred nanometers diameter may increase the bioavailability and extend blood circulation time of insoluble drugs [39,40,41]. According to the enhanced permeability and retention (EPR) effect, nanocarriers have passive targeting property (tend to enter tumors from irregular microvasculature and stay there for a longer period than small molecule drugs) [42,43]. Equipped with targeting ligands, nanocarriers are imparted with active targeting property (tend to actively accumulate in specific tumor tissues), reducing systematic toxicity and enhancing therapeutic effects [44,45]. Moreover, if triggered by external stimuli within tumor tissues or cells, nanocarriers may release the loaded drugs, directly exerting antitumor effects [43,46].

However, previous nanocarriers generally load one drug only [21], which may be less lethal to tumor cells, especially multi-drug resistance (MDR) tumor cells [22,23]. It is possibly difficult for these single-drug nanocarriers to inhibit recurrence or metastasis of tumors. In this review, we discuss double-cargo nanocarriers that deliver both small-molecule anti-tumor drugs and nano-sized photothermal materials.

### 2.1. Ingredients of Nanocarriers

According to our analysis for the literatures cited in this review, we found that all the nanocarriers have three-plus-two ingredients, i.e., three basic ingredients and two optional ingredients. The basic ones are anti-tumor drugs, photothermal materials and high molecular polymers, while the optional ones are the so-called drug-binding frames (see also Section 2.2.2) and targeting ligands.

Anti-tumor drugs. In all papers cited in this review, anti-tumor drugs that exerts chemotherapeutic effects on tumors are frequently doxorubicin (DOX), docetaxel (DTX) or paclitaxel (PTX) and sometimes cisplatin [47]. Other uncommon drugs may appear, e.g., resveratrol, SN38, AMD3100, curcumin, rapamycin, wedelolactone and so on [21,24,28,45,48,49,50,51]. Undoubtedly, DOX is the most common drug. In our opinion, the reason why DOX is so popular is as follows: (1) DOX is a kind of anti-tumor drug approved by the Food and Drug Administration (FDA); (2) DOX is so hydrophobic that it tends to disperse within the hydrophobic core of nanocarriers; (3) DOX has a polycyclic aromatic structure that may bind to 2-D materials e.g., graphene via π-π interactions. DTX and PTX, which are also FDA-approved, have multiple benzene rings that may also be employed via π-π interactions. The reports about other uncommon drugs should be just the preliminary explorations towards unprecedented drugs. Additionally, nucleotides sometimes may act as anti-tumor drugs and carry out gene therapy [52,53,54,55,56,57].

Photoabsorbers. A number of photoabsorbing materials, which produce heat for PTT, have been cooperated with drugs to obtain synergistic effects. The frequently used ones are nanogold, carbon-based materials tubes, poly dopamine nanoparticles, nano-sized Fe_3_O_4_, metal chalcogenide or dichalcogenide, indocyanine green (ICG) [41,47,58,59,60,61]. Nanogold materials may absorb photons and emit heat quickly and efficiently, because it has tunable and strong localized surface plasmon resonance (LSPR) [24]. Nanogold materials also have good bioavailability, low immunogenicity, controllable size and/or shape, easy surface modification [62]. The above properties should make nanogold more popular. Graphene oxide, with large loading capacity, ease for surface modification, satisfactory photothermal properties and planar structure for π-π interactions that may bind anti-tumor drugs such as DOX [11,63], is also frequently used on nano-sized drug delivery systems (DDS). Indocyanine green (ICG) and polydopamine (PDA), both of which are organic photoabsorbers that may be degraded in vivo [36], are also common.

Polymers. Generally speaking, the last basic ingredient, polymers, do not only bring bioavailability to nanocarriers owing to their good water solubility [47] but also alter the diameter of nanocarriers for utilizing the EPR effect [12,64]. Sometimes, the polymer serves as the container of drug and/or the photoabsorbers, e.g., polypyrrole (PPy) and polydopamine (PDA) [21,47,49,65,66,67,68,69,70,71,72,73]. Besides, anti-tumor drugs, e.g., curcumin or pluronic, may be integrated into polymers in some reports [19,37]. PEG is the most common polymer involved in this review. DNA may serve as good polymer, because DOX can be intercalated into DNA. DNA can bind to polyethylenimin (PEI) via electrostatic interaction. Wang et al. managed to load PEI with DOX-intercalated DNA and wrapped gold nanorods (AuNRs) with the polymer-based composite, fabricating a multi-modal nanocarrier with a very low IC_50_ of 2.88 µg/mL for MCF-7/ADR cells [74]. In some reports, unconventional polymers were also utilized, e.g., aptamer [60,75] and even red blood cell (RBC) membrane [76].

Targeting ligands. e.g., biotin, folate acid (FA) or cRGD, may lead to the active accumulation of nanocarriers within tumor tissues [21,45,77]. However, not all cases involved in this review utilize targeting ligands. Namely, they are optional. We consider them necessary to develop practical nanocarriers, though. Both in vitro and in vivo anti-tumor experiments may describe the differences between nanocarrier with and without targeting ligands. For example, the nanocarrier equipped with biotin (called DOX/ICG@biotin-PEG-AuNC-PCM) exhibited twice the cell inhibition rate (against MCF-7/ADR cells) of that of its counterpart without biotin, where biotin serves as the targeting ligand [78]. Another nanocarrier called GO@Gd-PEG-FA/DOX equipped with FA as the targeting ligand also had a DOX concentration of ~6 µg/g in liver, statistically significantly higher than that of the nanocarrier called GO@Gd-PEG/DOX without FA (~3 µg/g) [79]. The relative tumor volume of the GO@GD-PEG/DOX was 2.5 times as high as that of its counterparts with FA [79].

### 2.2. Core-Shell Structured Nanocarriers

Diverse platforms of nanocarriers have different structures, coupled with different preparation methods. According to their structures, we classify most of nanocarriers into two basic categories: core-shell structured ones and frame-coat structured ones.

**Figure 1:**
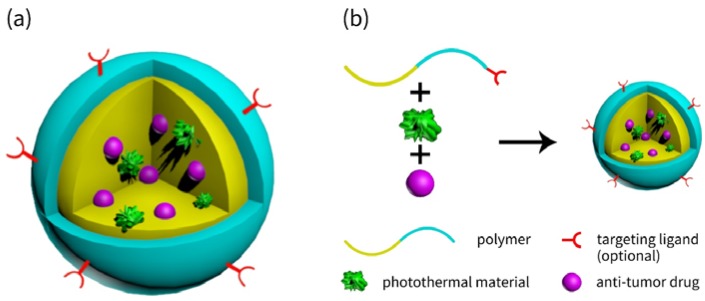
Schematic illustration of (**a**) the core-shell structured nanocarriers and (**b**) the preparation process of such nanocarriers.

Core-shell Structured Nanocarriers (Figure 1a) commonly have a hydrophobic core where drug molecules are encompassed and a hydrophilic shell which has a direct contact with the outside world, e.g., aqueous solutions or mice blood. Table 1 lists the references about these nanocarriers quoted in this review.

#### 2.2.1. Typical Core-Shell Structured Nanocarriers

**Figure 2:**
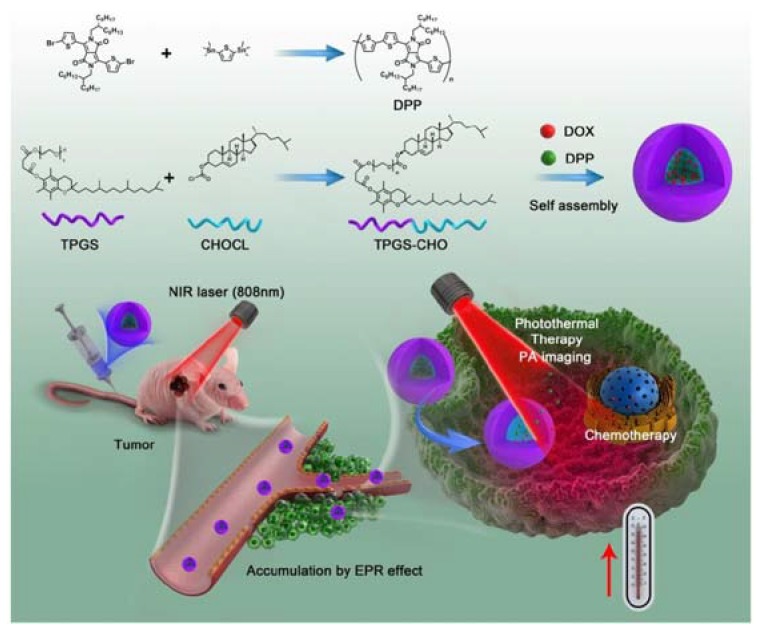
Schematic of the synthesis and photoacoustic imaging-guided photothermal/chemo combination therapy micelle. Reproduced with permission from [80]. The Royal Society of Chemistry, 2017.

Typical examples of this kind of nanocarriers are micelles, liposomes and nanohydrogels, whose cores and shells are composed of amphiphilic polymers, while the cores and shells of liposomes are phospholipids. By the way, common methods of preparing micelles or liposomes are also illustrated in Figure 1b. For example, Cao et al. prepared a typical micelle for co-delivery of DOX and DPP (the organic photothermal material used by the research team) [80]. The micelle was constructed by tocopheryl-polyethylene-glycol-succinate-cholesterol (TPGS-CHO) copolymers that embraces DOX and DPP within the hydrophobic core by self-assembly in water (Figure 2).

#### 2.2.2. Recent Advances of Novel Core-Shell Structured Nanocarriers

Traditional micelles and liposomes are so well-researched that they are easy to be prepared, characterized and tested, viz. lack of adequate creations. Nonetheless, some tiny innovations still appeared to improve the stability and/or in vivo performance of these nanoparticles. Several teams managed to construct micelles or liposomes with more than one amphiphilic polymers to utilize their properties to improve the properties of micelles [14,19,37,60]. Li et al. utilized three matters, phosphatidylcholine (PC), medium chain triglycerides (MCT) and trilaurin, to constitute liposomes for better stability and more space to accommodate cargoes [3]. Hong et al. employed up to four polymers to construct micelles, where Pluronic L61 unimer (one of these polymers) may reverse drug resistance of many type of tumors, another polymer called Phis-PLA-PEG-PLA-Phis may degrade at low pH outside or inside tumor cells [19].

On the other hand, there are some unconventional micelles with separate cores and shells [26,81,82,83]. Namely, the core and the shell of those micelles are originally different matters, respectively. Chemical bonds may form or not between the core and the shell during the preparation of nanocarriers. A smart drug delivery system (DDS) based on poly (lactic-*co*-glycolic acid) (PLGA) and polydopamine (PDA) was developed, wherein PDA was synthesized on the surface of previously fabricated PLGA nanoparticles [26]. Zhu et al. managed to use chitosan (CTS) as the shell to encapsulate docetaxel (DTX) and CuS-embedded povidone nanoparticles, preparing a micelle-like nanocarrier [81]. Zhong et al. constructed micelles with oleic acid-modified CuS nanoparticles (OA-CuS-NP) encapsulated in chitosan (CTS), where the hydrophobic oleic acid moiety and the hydrophilic CTS were linked via chemical bonds during the envelopment of OA-CuS-NP with CTS [82]. Hung et al. wrap hydrophobic PLGA that loads DOX and ICG with hydrophilic *N*-acetyl histidine modified d-alpha-tocopheryl polyethylene glycol succinate (NAcHis-TPGS), via the vitamin E moiety on the surface of the PLGA nanoparticle [83].

Some nanocarriers are reported to have improved in vivo performance. To evade unnecessary binding to negatively-charged serum protein but enhance successfully endocytosis into tumor cells with negative surface charges, Hung et al. constructed negatively-charged nanocarriers whose surface charge may be transformed to positive (or neutral) in the weak acidic environment outside cancerous cells, by introducing to nanocarriers the *N*-acetyl histidine moiety that may be protonated when pH < 7 [83]. Additionally, nano-in-micro particle spheres (NIMPS) that comprises micro-sized polymer-based spheres enclose the nano-sized moieties inside [23].

Moreover, there are also other examples of nanocarriers with unprecedented components. Modification of Prussian Blue nanoparticles (PBNPs) with oleylamine-toluene does not only changed the surface property of PBNPs for higher encapsulation efficiency but also provide new sites (phenyls in the toluene moiety) to bind DOX [27]. A team fabricated ultra-small sponge-like graphene nanosheets (graphene nanosponge) bearing a multiple of infinitesimal pores, wherein DTX and perfluorohexane (PFH) are stored [84]. PFH is gasified to eject DTX from the carrier on the heat produced through PTT, compelling drug release. Two teams employed cells as the source of the shells that pack drug and photoabsorbent: one team using red blood cells (RBCs) and the other using mesenchymal stem cells (MSCs) [76,93]. However, we are suspicious of the immunogenicity of both cell-based composite nanocarriers.

### 2.3. Frame-Coat Structured Nanocarriers

**Figure 3:**
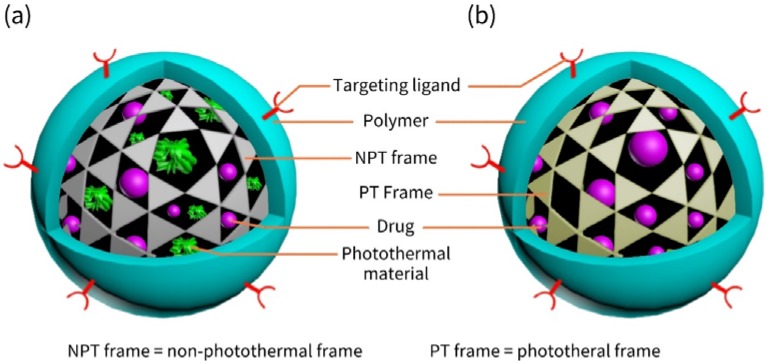
Schematic illustration of frame-coat structured nanocarriers. (**a**) Nanocarriers with a non-photothermal frame and photothermal materials; (**b**) Nanocarriers with a photothermal frame.

This kind of nanocarriers (Figure 3) tend to exist in diverse modalities but have common structural features. First, they have a drug-binding moiety which serves as interior supports just like human skeleton. We call it “frame.” Second, the frame is wrapped with hydrophilic or amphiphilic polymers, e.g., PEG or CTS, which bind to the frame via chemical bonds or just physical interactions. The water-solubility of the frame is hence increased by the polymers, the blood circulation time elongated and the bioavailability improved. The polymers that are the exterior wrapper act as coats that enclose the frame, just like muscles and skins wrapping the skeleton to compose a complete person. We therefore call them “coat.” Sometimes, both drug molecules and photothermal materials are enclosed within the non-photothermal frame (NPT frame) that does not convert photon energy into heat (Figure 3a). In other cases, the photothermal frame (PT frame), which serves as photoabsorbers and emits heat, is used. (Figure 3b). Table 2 displays the articles cited in this paper that discuss such kind of nanocarriers.

#### 2.3.1. Various Frames

Frames are various in chemical composition and shape. They have only one common ground: drug-loading frames are wrapped by coats outside.

In the scope of this review, frames are (but not limited to) carbon-based nanomaterials (mesoporous carbon nanoparticle, graphene, graphene oxide, single- or multi-walled carbon nanotubes, etc.) [29,36,37,39,41,59,63,66,68,79,94,95,96,97,98], silica-based nanomaterials [30,31,93,99], nanogold materials [75,77,78,99,100,101,102,103], polydopamine (PDA) [28,47,67], polypyrrole (PPy) [27,49,69,70,71,72,73,94], metal organic frameworks [73,104], Prussian blue [104,115], other inorganic materials [22,35,69,105,106,107,108] and other uncommon organic materials [11,70,109,111]. Among these frames, photothermal frames are predominant. Non-photothermal frames are only (but not limited to) pure silica-based materials [30,31,93,99], polyethylenimine (PEI) [74], monoolein [11], nanogel G [109], CTS [110], etc.

It is noteworthy that some teams tried to use composites as frames. Zhang et al. prepared a nanocarrier with the frame as hollow mesoporous silica/carbon nanoparticle (Si/C NP) loading DOX and modified with PEG [39]. Wang et al. synthesized multi-walled carbon nanotube (MWNT) coated with PPy decorated with gold, trying to improve the photothermal conversion efficiency of MWNTs with PPy and gold [94]. Li et al. coated gold onto SiO_2_ nanospheres to construct the inner support of the nanocarriers, which were prepared by wrap SiO_2_@Au with polymer-modified graphene oxide [99]. Chen et al. utilized PPy confined within dendrimer-like silica nanoparticle (DSN) to construct their frames [71]. Li et al. integrated three chemicals into their frames [111]. Meng et al. even synthesized a complicated gel called **G** to serve as their frames, whose monomers are oligo (ethylene glycol) methacrylate (OEGMA) and 2-(2-methoxyethoxy) ethyl methacrylate (MEO2MA) [109].

Some reports introduced the same chemical as the frame and the coat [110], e.g., Gao et al. using CTS [110], Chen et al. using gold nanostar (AuNS) [103], Hou et al. and Zhang et al. using graphene oxide (GO) [63], Wang et al. using PDA [28], Park et al. using PPy [72], Gao et al. using carbon nanotubes (CNTs) [96], etc.

On the other hand, not all nanocarriers involved in this review are spherical, viz. polymorphism. The most typical examples are diverse nanogold-based frames, including nanostars, nanorods, nanoplates and nanocages [75,77,78,100,101,103]. Other 2-D materials-based frames were utilized for relatively flat nanocarriers, e.g., PEI [74], ZnO nanosheets [35], nano graphene (or graphene oxide) [36,59,63,66,68,79,97,98] etc.

However, there still exist some nanocarriers designed with novel ideas that are distinctive from the ones mentioned above. Two teams independently prepared nanocarriers whose frame and coat are swapped, respectively [73,104]. In both reports, the “reversed” nanocarriers comprise of hydrophobic polypyrrole (PPy) as the inner support and hydrophilic Mil-100(Fe) as the outer coat. Surprisingly, it is the outer Mil-100(Fe), not the inner PPy, that binds and discharges anti-tumor DOX. Likewise, Wang et al. fabricated similar “swapped” nanocarriers with Prussian blue nanoparticles (PB-NP) as the frame and Mil-100(Fe) as the coat [107]. Among the three reports, the inner frame (PPy and PB-NP) are all responsible for PTT.

#### 2.3.2. Preparation of Frame-Coat Structured Nanocarriers

**Figure 4:**
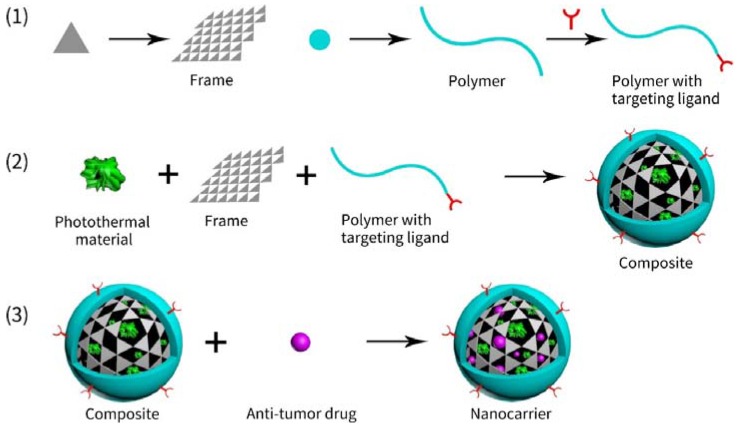
Schematic illustration of preparing frame-coat structured nanocarriers.

The process of preparing these nanocarriers may generally have three steps (see Figure 4): (1) individual manufacture of different components (frame and polymer), (2) encapsulation of frame with polymers and/or photoabsorbers to form completely functional nanoparticles, (3) combination of unloaded nanocarriers and drugs (and photoabsorbers, if exist). Notes: (1) The 2nd step and 3rd step are often swapped; (2) Every step may be composed of several smaller steps (chemical reaction and/or physical processing).

**Figure 5:**
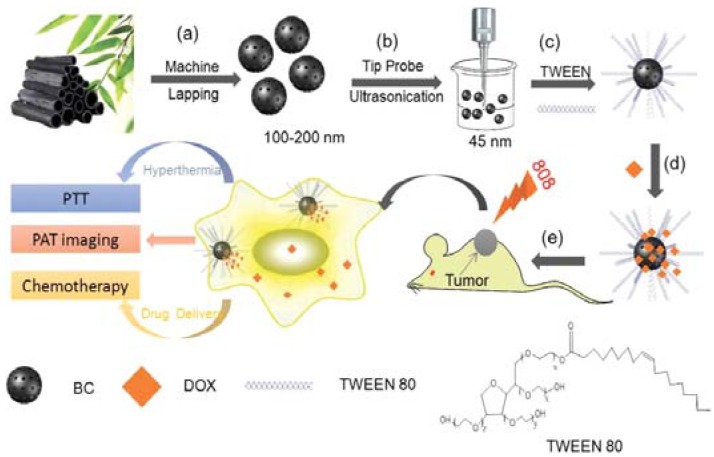
Schematic illustration of (**a**,**b**) experimental design for the synthesis of BCNPs; (**c**,**d**) BCNPs functionalized with TWEEN 80 for DOX loading; (**e**) BCNPs as a novel theranostic platform for PAT imaging-guided 808 nm NIR-laser-stimuli-responsive chemo-photothermal therapy of tumor. BCNP = bamboo charcoal nanoparticle. Reproduced with permission from [85]. Wiley, 2016.

For example, Dong et al. prepared a nanocarrier based on bamboo charcoal nanoparticles (BCNPs) [85]. The steps are depicted in Figure 5. The first step is to prepare BCNPs through machine lapping and then ultrasonication (Figure 5a,b). The second step is to modify the surface of BCNPs with TWEEN 80, a high-polymer surfactant (Figure 5c). The third step is to load DOX to BCNP/TWEEN 80 composite (Figure 5e). According to the authors of this paper, the DOX-BCNP/TWEEN 80 may exert in vivo synergistic effects of CHT + PTT under NIR radiation after intravenous injection into mice.

A similar example is that Li et al. prepared a nanocarrier by modifying mesoporous carbon nanoparticle (MCN) with poly(ethylene glycol)–poly(curcumin-dithiodipropionic acid) (PEG-PCDA) [37]. The first step was to synthesize MCN via the low-concentration hydrothermal route and to purify MCN by H_2_SO_4_ (98%). The second step was to load DOX to MCN by physical process—sonication and stirring. The third step was to modify the surface of DOX@MCNs with PEG-PCDA by the thin film-ultrasonic dispersion method.

Another example with a little more complexity was hollow ZrO_2_/PPy nanoplatform loading DOX [69]. The first step was to synthesize hollow ZrO_2_ nanoparticle by the growth of ZrO_2_ on the surface of SiO_2_ nanospheres and the following etching of SiO_2_ by 0.1 M NaOH at 80 °C. The second step was to synthesize PPy confined within hollow ZrO_2_ nanoparticles by removing PPy outside the hollow nanoparticles after polymerization of pyrrole. The third step was to load DOX to the hollow ZrO_2_/PPy nanoplatforms.

A much more complicated example is a nanoplatform called NRGO-GNS@DOX nanocomposites [66]. The first step was to prepare nanosized reduced graphene oxide (NRGO), dopamine (DA), gold nanostars (GNS) and HS-PEG (PEG terminated with a sulfhydryl group). The second step was to wrap NRGO with polydopamine (PDA), GNS and PEG-SH. PDA was synthesized on the surface of NRGO via the polymerization of DA. GNS were then mounted onto the PDA layer on NRGO via Au-catechol interactions. PEG-SH was further conglutinated to GNS via the Au-S bond. The third step was to load DOX to the NRGO-GNS via the π-π interactions between DOX and NRGO.

**Figure 6:**
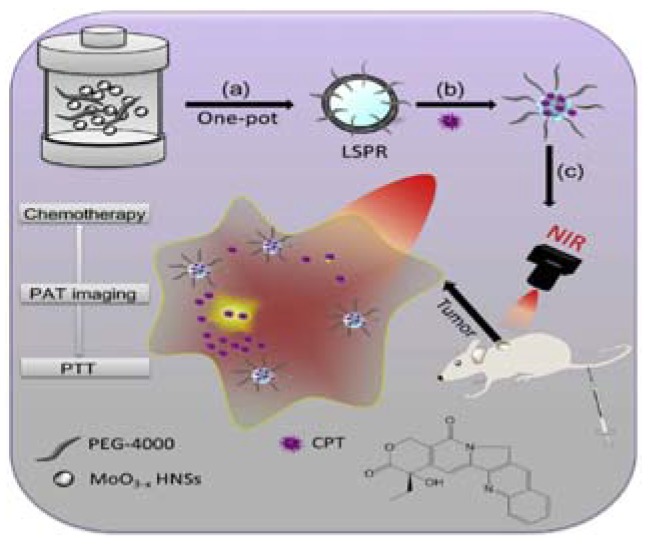
Schematic illustration of (**a**,**b**) the synthesis process of CPT loaded PEG-MoO_3−*x*_ HNSs nanovehicle for (**c**) PAT imaging-guided combinational chemo-photothermal therapy of cancer. Reproduced with permission from [106]. Elsevier, 2016.

Exceptional examples still exist, though. Not all frame-coat structured nanocarriers are prepared following the three steps above. As shown in Figure 6, the one-pot synthesis method is applied to prepare PEGylated oxygen deficiency molybdenum oxide hollow nanospheres (PEG-MoO_3−*x*_ HNSs) [106]. PEG-MoO_3−*x*_ HNSs were simply prepared by hydrothermal process performed on fully mixed [(NH_4_)_6_Mo_7_O_24_·4H_2_O] and PEG-4000. The loading of camptothecin (CPT) to PEG-MoO_3−*x*_ HNSs was implemented by stirring in the dark. One other interesting example is a gold nanostars-based nanocarrier prepared by coating the frame (gold nanostars) with several layers of coats, including two mitochondria-targeting peptides and hyaluronic acid (HA) [103].

### 2.4. Navigation of Nanocarriers to Tumors

There are two common ways to increase active accumulation of nanocarriers: (1) addition of targeting ligands and (2) application of external magnetic field. Targeting ligands may bind to specific sites on the surface of cancer cells, promoting nanocarriers entrance into tumor tissues [21]. Occasionally, antibody may serve as targeting ligands, adding some anti-tumor capacity to nanocarriers, e.g., human epidermal growth factor 2 (HER-2) antibody against breast cancer [9,61]. External magnetic field may guide nanocarriers with a magnetic moiety (the most common one is Fe_3_O_4_) to the tumor [112]. Both ways will help concentrate drug molecules and photothermal materials within tumor tissues, leading to further enhanced antitumor effects and more reduced systematic side effects than the nanocarriers without targeting ligands or magnetic assistance.

### 2.5. A New Trend: Preventions to Premature Leak of Drug

It is also important to prevent premature leak of drug from nanocarriers in physiological or pathological environment before they reach target tumors [35]. If these nanocarriers are injected into patients, they would flow within human blood circulation for a long time, during which the interactions between drug molecules and nanocarriers may be loosened or even disrupted. Porous nanovehicles (e.g., mesoporous silica nanoparticles, hollow polypyrrole nanospheres) or flat nanovehicles that carries drug via non-chemical interactions (e.g., graphene, nanogold, etc.) are subject to the tendency of loose connections [69,99,102,103,116]. Drug molecules might disengage from the loose connections and pass into blood before the nanocarriers enter tumors, causing systematic side effects, though not too serious. Given those aspects above, some scientists tried to seal nanocarriers with special hindrances. Huang et al. tried to block the small pores in gold nanocages (AuNCs) with hyaluronic acid (HA) that encapsulated DOX for fear of leakage [101] but HA is so unstable and hydrophilic that it may not be adequate. Compared with HA, some phase-change materials are more competent. For instance, tetradecanol (TD), whose melting point is between 38 and 39 °C, may enclose DOX at body temperature (37 °C) or blow but may disgorge DOX when PTT is performed (~42 °C), thus TD was utilized to prevent premature drug release [77,78]. Likewise, pentadecanol was employed to trap DOX within hollow magnetic Fe_3_O_4_ nanoparticles coated by Prussian blue (PB) in another report [115]; but we are suspicious of the bioavailability and biocompatibility of the nanocarrier since PB is not water-soluble. Measures to avoid premature drug leak from the nanocarriers is a new trend that appeared since early 2017. Provided that nanocarriers spend a long time in blood circulation, it is necessary to enclose drug tightly within carriers to reduce systematic side effects caused by escaped drug.

## 3. Combination of Chemotherapy with Photothermal Therapy via Nanocarriers

### 3.1. The Basics of Photothermal Therapy

Photothermal therapy (PTT), a treatment that external near-infrared (NIR) light is converted to heat by photothermal materials to destruct adjacent cells or tissues by increasing the temperature, has attracted increasing attention in anti-cancer research [24,25]. NIR light tends to penetrate deeper to human body because of its optical property and rare absorption by human tissues in its way [79,117]. The light hence does little harm to non-cancerous cells or tissues before it is mainly absorbed by photoabsorbers, viz. NIR light is little or no invasive [39,118]. Consequently, PTT displays many features, e.g., deeper penetration, less invasiveness, no DNA damage, no water absorption [7,119]. PTT has high selectivity and low side effects. Irradiated by NIR light, photothermal materials absorb light and emit heat, causing hyperthermia (rising temperature) around them [24]. When the temperature is between 41.8 and 45 °C, tumor tissues are impaired because cells within hypoxic (low oxygen) tumors are subject to heat [120], thus making possible tumor ablation. However, healthy tissues are little affected in the temperature range [120]. PTT thus has infinitesimal side effects [115]. If NIR light is cast on tumors where photothermal materials are specifically delivered, PTT exhibits high selectivity: hyperthermia largely affects tumors while exerts little damage to other tissues [60]. Photothermal materials suitable for therapy should have low toxicity, high photothermal conversion efficiency and the potential to accumulate within tumors [3]. However, owing to the limited radiant power, unsatisfactory penetration depth and inhomogeneous heat distribution in tissues, PTT per se is not always able to completely remove tumors [26,27].

### 3.2. The Interactions of Chemotherapy and Photothermal Therapy

In principle, the synergistic effect is achieved by simultaneously affecting multiple pathways of the same disease with various therapies [3]. PTT and CHT may perform well synergistic effect through cooperation. Firstly, PTT tends to ruin tumor cells directly and physically, leading to cell apoptosis or necrosis [24,66]. Usually, NIR radiation, which penetrates skin and other tissues in the way of the light beam, is converted by photothermal materials located in tumor tissues to heat, causing hyperthermia wherein thermo-sensitive tumor cells are likely to be damaged and tumors to be ablated. Secondly, CHT impairs tumor cells through chemical or biological pathways, in our opinions. For example, DOX (the most frequently reported anti-tumor drug in articles quoted in this review) does not only prevent DNA duplication via intercalation into DNA molecules but also generate free radicals to damage cell structures (esp. mitochondria) and reduce cell apoptosis [9,29,66]. Thirdly, mild hyperthermia (<43 °C) caused by PTT may exert on tumors many influents which raise cellular uptake of drug and enhance anti-tumor effects of CHT: quickening blood steam near the focal, damaging tumor vasculature, compelling nanocarriers extravasation from the vasculature, improving drug entrance into tumors, increasing membrane permeability, gaining tumor cell sensitivity to CHT, accelerating cellular metabolism and inducing possible anti-cancer immune responses [12,24,27,71,86,96,105]. Fourthly, CHT may complement PTT by destroying tumor cells that escaped PTT and vice versa [26,28,72]. Heat-resistant tumor cells and drug-resistant ones are, respectively, cleared by CHT and PTT in shorter time than does individual therapy alone. Subsequently, relapse and metastasis may be hindered. Given the above, the combined therapy is not a simple pile of CHT and PTT but a union that matches them up to give rise to synergistic effect [31].

### 3.3. The Ways to Fight Against Multi-Drug Resistance

Multi-drug resistance (MDR), the leading reason to tumor relapse, is mainly related to the overexpression of ATP-binding cassette (ABC) transporters, e.g., P-glycoprotein (P-gp), on cell membrane, which facilitate the efflux of drugs from tumor cells [19]. Correspondingly, PTT decreases the expression of P-gp to prevent DOX efflux, making MDR tumor cells more sensitive to DOX (and/or other anti-tumor drugs) [86]. Moreover, PTT induces expression of the heat shock protein (HSP) trimer and reduces the expression of mutant p53, further enhancing the therapeutic effects [86]. One the other hand, MDR is also related to the cell survival pathways such as the phosphoinositide-3-kinase (PI3K)/Akt mechanism, which may be inhibited by rapamycin (RAPA); a combinatory therapy based on liposomes wrapping DOX, RAPA and photoabsorbent is accordingly reported by Thapa et al. [51].

### 3.4. Light-Controlled Drug Release

Light-controlled drug release is a distinctive feature of the double-cargo nanocarriers. For accurate release of drug within tumors, stimuli-responsive drug delivery systems are developed and prevalent [87], which are triggered to release drugs by specific stimuli accompanying tumors, e.g., low pH, high glutathione (GSH) level or reactive oxygen species (ROS) [14,28]. As is known, the simultaneous presence of drug release and NIR radiation may obtain the maximum therapeutic effects. Nonetheless, stimuli-triggered nanocarriers may unload drug molecules before or after PTT [121]. It is hence necessary to adjust drug release to synchronize CHT with PTT. Given that photothermal materials may emit heat and generate reactive oxygen species (ROS) under NIR light, scientists may integrate heat-responsive or ROS-responsive components into nanocarriers. When external NIR light is casted on thermo-responsive nanocarriers encapsulating drugs and photothermal materials, the heat or ROS compels the nanocarriers to unload drug molecules, making drug release controllable and predictable [88,112]. Common nano-vehicles may discharge their cargoes because the heat accompanying PTT loosens the interactions between carriers and drug molecules (e.g., the π-π interactions between DOX and carbon-based nanomaterials [11,96]), or because ROS disrupts the structures of carriers (e.g., the lipid moiety [50,88]). Zhang et al. devised a liposome whose membrane phase will be transformed from gel to crystalline phase by the PTT-induced hyperthermia, viz. a bulk release of drug upon NIR irradiation, enhancing anti-tumor effects [50]. It is noteworthy that three teams developed nanogels that shrink to squeeze DOX out of the carrier at high temperature during PTT with different heat-responsive polymers, respectively [20,34,111]. Additionally, some reports [28,37,74,77,101] shows that nanocarriers are further designed to discharge drug if triggered by more than three external stimuli to ensure more accurate release, for multiple stimuli ensure the accurate and synchronous release of anti-tumor agents.

## 4. The Therapeutic Effects of Combinational Therapy with Nanocarriers

How to clarify the synergistic effect of CHT and PTT by experiments? According to the analysis of all papers we retrieved, we found a fact: the more experiments, the more persuasive. Generally, both in vitro and in vivo experiments are necessary. A paper on nanosized drug delivery system without in vivo experiments is not intact and less persuasive. Consequently, papers including detailed and comprehensive experiments containing both in vitro and in vivo ones tend to be published in high IF journals. Among these experiments, we focused on in vitro anti-tumor effect assays (to evaluate the toxicity of drug-loaded nanocarriers coupled with NIR radiation by determining cell viability) and in vivo anti-tumor effect assays (to evaluate the ability of drug-loaded nanocarriers coupled with NIR radiation to inhibit solid tumor growth by determining relative tumor volume). Both assays offer the most convincing data that describe the synergistic anti-tumor effect. If the combined therapy of PTT + CHT efficiently exerts more anti-tumor effect than PTT alone or CHT alone does, the conclusion is tenable that the synergistic effect does exist.

### 4.1. In Vitro Cell Experiments

All papers listed in Table 1 and Table 2 covered in vitro cell experiments. Potential drug-loaded nanocarriers are supposed to display high cytotoxicity under NIR radiation against tumor cell lines. High cytotoxicity is quantitatively reflected by low cell viability or high apoptosis rate. MTT assay is the most common method to determine cell viability though, similar methods such as CCK-8 assay are acceptable. Moreover, flow cytometry is the most common method to determine apoptosis rate. Given that the prerequisite of therapeutic effect is the entrance of nanocarriers into cells, fluorescent microscopy is a useful tool to reveal whether nanocarriers are swallowed by tumor cells by observing the fluorescent emitted from within cells. On the other hand, fluorescent microscopy may intuitively display the distribution of live or dead cells. It is noteworthy that the combined therapy of PTT and CHT will efficiently inhibited (or even almost eliminate) tumor cells, while neither of PTT alone nor CHT alone does.

**Figure 7:**
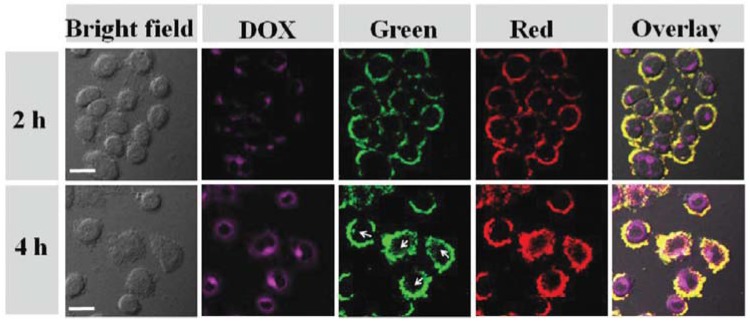
Confocal laser scanning microscopy (CLSM) images of HeLa cells after incubation with UCNPs@Au-DOX for 2 and 4 h, respectively; the rose red emission from DOX was collected at 550–700 nm under excitation of 488 nm; upconversion luminescence (UCL) emission was collected by a green UCL channel at 500–600 nm and a red channel at 600–700 nm, λ_ex_ = 980 nm, 500 mW. UCNP = upconversion nanoparticle. UCNPs@Au-DOX is the name of a nanosized drug delivery system. Reproduced from [122] with permission from The Royal Society of Chemistry.

Several examples are as follows. As shown in Figure 7, for example, the fluorescent of DOX (rosy) and the upconversion luminescence (UCL) emission (green and red) were collected by a fluorescent microscope, respectively [122]. The overlay pictures indicate that all fluorescence and luminescence came from the inner cells. The CCK-8 assay showed that the viability of Hela cells treated with UCNPs@Au-DOX and laser (CHT + PTT) was almost 0 at the utmost concentration of the nanocarriers, which was significantly lower than that of cells treated with PTT only (~20%) and nanocarrier + DOX (~70%). Namely, the synergistic effect is achieved.

Another example illustrated the preparation of the arylboronic ester and cholesterol modified hyaluronicacid (PPE-Chol_1_-HA, PCH) nanoparticles for loading DOX and ICG [88]. In the article, fluorescence photos were used to evidence that the nanocarrier had managed to enter tumor cells. MTT assay was performed to compare the cell inhibition rate of different formulations of nanocarriers. At the utmost concentration of ICG (16 µg/mL) and DOX (20 µg/mL), the PCH-DI/Laser (CHT + PTT) group had the utmost inhibition rate (~90%), significantly higher than that of the PCH-I/Laser (PTT only) group (~50%) and the PCH-D (CHT only) group (~65%). Flow cytometry further quantitatively showed the in vitro synergistic anti-tumor effects of the nanocarrier and the apoptic effect of the arylboronic ester (P) moiety. The PCH-DI/Laser group had the least live cells (44.31%), lower than that of the PCH-DI group (69.71%) and CH-DI group (52.57%).

After the administration of multi-layered liposome to HeLa cells, Wang et al. found that the endocytosis was not related to NIR radiation using fluorescent microscopy [24]. With a series of MTT assays, Wang et al. observed the dose-dependent anti-proliferative effects of the nanocarrier against HeLa cells, described the effects with several diagrams and affirmed the synergistic effect of CHT and PTT. After all, in vitro cell experiments are necessary for researchers to exhibit the ability of their nanocarriers to intracellularly fight against tumor.

### 4.2. In Vivo Experiments

The most important experiment is the tumor inhibition assay where the tumor volume and tumor weight are determined at intervals and the relative tumor volume (RTV) is calculated. The experiment directly and intuitively shows the therapeutic effects. Additionally, the biodistribution is necessary to indicate the accumulation of nanocarriers within tumors. Other experiments, including in vivo photothermal effects, bodyweight, H&E staining, are needed to describe the health status of mice from different perspective. The lower RTV, the better therapeutic effects. Several examples are listed below.

**Figure 8:**
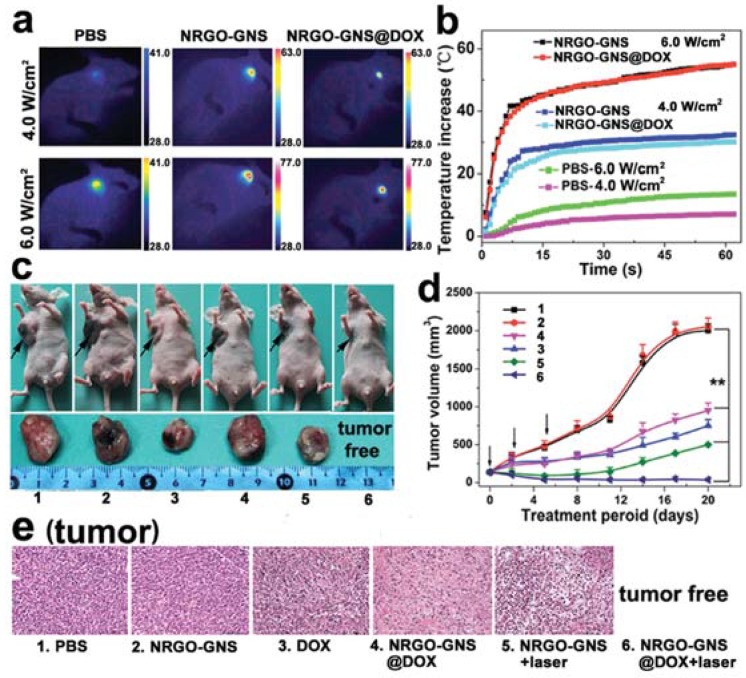
(**a**) IR thermal images of PBS-, NRGO-GNS-, or NRGO-GNS@DOX-injected 4T1 tumors after laser illumination; (**b**) Photothermal effect of PBS, NRGO-GNS, or NRGO-GNS@DOX in orthotopic 4T1 tumor-bearing mice in vivo; (**c**) Representative photographs of the tumor-bearing mice (top panel) and the tumor tissue (bottom panel) taken at the 20th day post the first treatment (the black arrows indicated the position of the primary tumors); (**d**) Tumor volume change examined during the therapeutic period (*n* = 6, ** *p* < 0.01, compared to PBS injected mouse controls); (**e**) H&E staining of the tumor sections at the end of antitumor study (200×). NRGO = nanosized reduced graphene oxide. GNS = gold nanostar. Reproduced with permission from [66]. Wiley, 2016.

Wang et al. prepared a nanocarrier called NRGO-GNS@DOX composites and preformed a set of in vivo researches (Figure 8), where NRGO is nanosized reduced graphene oxide and GNS represents gold nanostars [66]. (1) Photothermal effects experiments where the temperatures of the sites under NIR illumination with different powers were determined, photographed (Figure 8a) and plotted (Figure 8b). The temperature exceeds 40 °C at 6.0 W/cm^2^ in just 8 min. (2) in vivo tumor inhibition experiment where tumor volumes were recorded, RTV were calculated and 4T1 tumor-xenografted mice coupled with their corresponding tumors at the 20th day were photographed (Figure 8c,d). After a therapeutic period of 20 days, no primary tumors were found in all 4T1 tumor-bearing mice treated with the combined therapy. In contrast, the average tumor volume of the mice treated with PTT alone and CHT alone was ~500 and ~1000 mm^3^, respectively. The data indicated that the synergistic effect is obtained and satisfactory. Additionally, no metastasis foci were found in the PTT + CHT group of mice, indicating the complete eradication of in situ solid tumors. (3) The H&E staining of tumor tissues (Figure 8e) from every group intuitively illustrated the damage of therapies on tumors.

**Figure 9:**
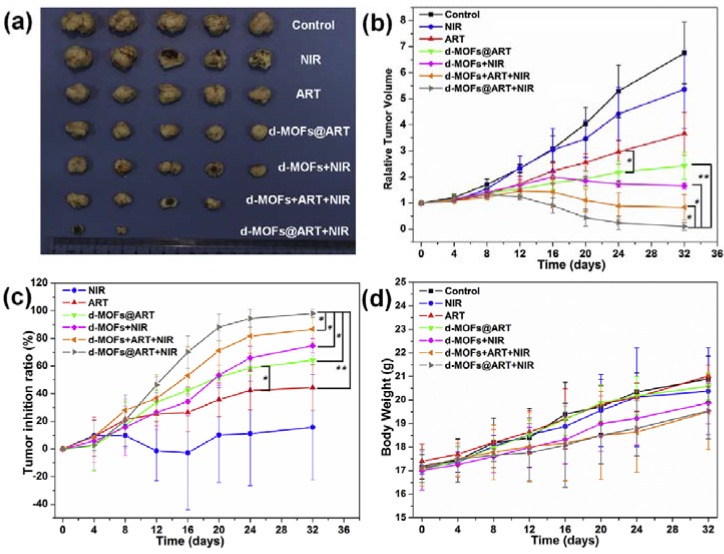
Antitumor ability of ART-loaded d-MOFs nanospheres in HeLa xenograft mice models. (**a**) The excised tumors from different mice groups treated with saline, NIR irradiation, free ART, d-MOFs@ART, d-MOFs + NIR, d-MOFs + ART + NIR (non-encapsulated) and d-MOFs@ART + NIR at 32nd day; (**b**) Tumor growth curves of mice after various treatments (mean ± SD., *n* = 5, * *p* < 0.05, ** *p* < 0.01); (**c**) Tumor inhibition ratio of the NIR irradiation, free ART, d-MOFs@ART and d-MOFs + NIR group and d-MOFs@ART + NIR tumor inhibition ratio of chemo-thermal therapy (mean ± SD., *n* = 5, * *p* < 0.05, ** *p* < 0.01); (**d**) Body weights of mice for varied time periods for 32 days after various treatments. ART = artemisinin. d-MOFs = dual metal organic frameworks. Reproduced with permission from [107]. Elsevier, 2016.

Another research team performed a handful of similar experiments to study their novel nanocarrier called d-MOFs@ART (Figure 9), which is an artemisinin (ART)-loaded nanoparticle with dual metal-organic-frameworks (d-MOFs), with Prussian Blue as the inner MOF and MIL100(Fe) as the outer MOF [107]. (1) Photothermal effects were not presented in Figure 9. (2) in vivo tumor inhibition experiment where RTV and tumor inhibition ratio were calculated according to tumor volumes determined every 4 days (Figure 9b,c) and the tumors excised from mice at the last day were photographed (Figure 9a). These results indicated that the d-MOFs@ART + NIR (CHT + PTT) group of mice had their tumor almost eradicated and the therapeutic effect of the group was significantly lower than that of the CHT alone group and the PTT alone. P.S. d-MOFs + ART + NIR represents the simply mixture of d-MOFs and ART, while d-MOFS@ART + NIR means ART is encapsulated within the carrier. (3) The smooth increase of the bodyweight of every group (Figure 9d) indicated that d-MOFs@ART does no harm the healthy tissues. (4) H&E staining of organs including heart, liver, spleen, lung and kidney were performed to show that d-MOFs@ART did not impair non-tumor organs (not presented in Figure 9). Xie et al. tried to modify curcumin (CUR)-loaded bamboo carbon nanoparticles (BCNPs) with D-α-Tocopherol PEG 1000 succinate (TPGS), obtaining a nanocarrier called TPGS-BCNPs@Curcumin [48]. After 16 days of treatment, the TPGS-BCNPs@Curcumin + NIR group of BEL-7402 tumor-bearing mice had their tumors almost eradicated (tumor growth inhibition: 99.75%) and had no recurrence. Similarly, TWEEN 80-modified BCNP loading DOX almost inhibited the xenografted tumor of BEL-7402 tumor-bearing mice under NIR radiation, with the inhibition rate of ~96% [85]. Besides, we noted several articles reporting a complete suppression or almost eradication of xenografted tumors [36,41,64,66,69,84,88,89,102,109].

### 4.3. Fighting Against Recurrence and Metastasis

Many nanocarriers with NIR radiation did not only almost eradicate in situ solid tumors but also managed to suppress recurrence during the period of treatment and observation [36,41,64,66,69,84,88,103,106,109]. These results indicated that the synergistic therapeutic effect is thorough and plenty. On the other hand, several research teams discussed the effect of their nanocarriers and NIR radiation on metastatic tumors. Several examples are listed below. Wang et al. reported that their nanocarrier, NRGO-GNS@DOX, eliminated both recurrence and metastasis in 20 days of treatment [66]. Thapa et al. synthesized a nanocarrier called PEG-GO/LCN/DTX with which metastatic DU145 cells were treated [11], where LCN is liquid crystalline nanoparticle and DTX is docetaxel. The cell viability of the PEG-GO/LCN/DTX + NIR group was less than 20%. The cell migration of the combined therapy group was almost 100%. Western blotting showed the combined therapy group had highly increased levels of apoptotic protein (p53 and bax) and cell cycle arrest markers (p21 and p27) compared with the DTX group alone, suggesting that the anti-metastatic effects are related to actions on apoptosis and cell cycle arrest. A complicated nanogel with the code name “**G**” was administered to SMMC-7721 tumor-bearing mice, causing that the **G** + DOX + CuS + NIR group had the least tumor metastasis nodules (~20) in mice livers at the 7th day after the first injection, significantly lower than other groups [109]. However, the number of papers mentioning anti-metastatic effects is limited. We infer that one possible mechanism is that most of in situ tumor cells are killed before MDR is induced and/or massive metastasis takes place.

### 4.4. Triple-Modality Therapies

Gene therapy (GNT) has also been introduced to construct triple therapy. combinational therapy: triple therapies based on GNT + CHT + PTT have been explored. A multifunctional aptamer AS1411, which is a guanosine-rich hairpin-like single-stranded DNA, may load up DOX via intercalation and bind to nucleolin that is expressed on the surface of or within the cytoplasm of cancer cells [75,123]. Brann et al. modified gold nanoplates (GNPs) with AS1411 equipped with DOX, obtaining a trimodal nanomedicine with GNP for PTT, AS1411 for GNT and DOX for CHT [75]. Yi et al. constructed a multimodal therapy called DT/ds-NRs with or without aptamer AS1411, which allows a union of GNT, CHT, PTT and photodynamic therapy (PDT),where Hela cells treated with DT/ds-NRs plus AS1411 demonstrated a much higher cytotoxicity of 7.7% than that of the same cells treated with DT/ds-NRs alone (48.6%) [100].

### 4.5. Pending Clinical Trials

While it is true that any polymer-based therapies containing nanoparticles are available in market or progressing in clinical trials [124,125], it is also true that, to our farthest exploration at NIH National Cancer Institute and ISI Web of Knowledge, we found no clinical trial reports on the double-cargo nanocarriers that performs both CHT and PTT against cancer. Additionally, according to our retrieve at NIH National Cancer Institute, a PTT nanoparticle named AuroShell^®^ is recorded (ClinicalTrials.gov Identifier: NCT02680535) but not yet completed. In our opinion, there are three main reasons to the phenomenon above. First, the entire process of the clinical trial is long (about 10–15 years) and expensive (about $1 billion) [125]. Second, nanoparticles (containing nanocarriers) often have so excessively complex formulations and structure that it is excessively difficult for researchers to demonstrate substantial equivalence to previously FDA-approved therapies [124]. Third, so many single-cargo nanocarriers that loads only one anti-tumor drug is in the process of clinical trial, not to mention the more complex double-cargo nanocarriers that perform CHT and PTT. In summary, the translation of laboratory-made nanocarriers to clinical trial is an unprecedently arduous process.

## 5. Problems, Hurdles and Challenges

Although researches on combined therapy of PTT and CHT through polymer-based composite nanocarriers have much progressed, there are still many hurdles that hinder clinic applications and challenge scientists to future studies. The hurdles include but not limited to, the following ones. Firstly, the barriers to extravasation from vasculature to tumor tissue should be further overcome for better accumulation. For example, the pea-like nanocomposites designed according to hydrokinetics moves faster in blood and have easier access to tumor tissues [64]. A liposome based on sponge-like nano-sized graphene could load DOX and perfluorohexane (PTH), the gasified PTH may eject DOX from the nanocarrier to tumor tissues when PTT makes graphene emit heat [84]. However, not all nanocarriers are so subtle and facile. Secondly, long-term toxicity of non-degradable and non-biocompatible photothermal materials, for example, the aforementioned photo-absorbers including MWNT, rGO, PPy, PDA, AuNR, AuNP, Fe_3_O_4_, etc., need to be further clarified [28]. Thirdly, photo-stable nano-materials with higher photothermal conversion efficiency, like gold and Bi_2_O_3_ might be the desired candidates for better therapeutic effects [97]. Fourthly, the likeliness of complicated multi-moiety nanocarriers to bring about new in vivo side effects should be taken into consideration [28]. For example, loose nanocarriers that have no or little stable links among moieties may be subject to disintegration of components or premature leakage of cargoes. Fifthly, the inhomogeneous hyperthermia expanding too large may bring collateral burns to tissues near tumors during NIR radiation [96]. Sixthly, industrial production of nanocarriers may encounter knotty engineering problems [126]. Some teams have tried scale-up, though [127,128]. However, there were no report of industrial production of the double-cargo nanocarriers to our farthest exploration of literature. We think it is difficult to comprehensively evaluate pilot-scale or large-scale production. Seventh, commercially available targeted therapies, e.g., sorafenib, temsirolimus, nivolumab, etc., may be the most potent competitor against nanocarriers [13]. In summary, we anticipate that above-mentioned problems should be addressed and clinically-feasible nanocarriers should be developed in the future.

## 6. Discussions and Suggestions

For ingredients, we classified various ingredients to three-plus-two categories. We suggest that DOX and a targeting ligand (e.g., FA) should be employed to cooperate with a new photoabsorber or a new polymer for the construction of a novel nanocarriers.

For micelles and/or liposomes, we anticipate that brand-new polymers are developed to prepare novel nanocarriers. According to our analysis for papers cited in this review, multi-stimuli-responsive polymers are welcome. However, the possible disintegration in vivo has severely compromised the potential applications of micelles and/or liposomes.

For frame-coat structured nanocarriers, carbon-based and silica-based frames are most popular. Serving both as the frame and the photoabsorber, PPy is also favorite to many research teams. We suggest that pure carbon-based materials (e.g., graphene and graphene oxide) and PPy should be used to construct novel nanocarriers that delivers new anti-tumor drugs or new polymers (new coats). In our opinion, silica-based frames (e.g., mesoporous silica nanoparticles) are also good choices. We also regard PPy as a bioavailable material which could facilitate the modification of new non-photothermal frames for the fabrication of novel nanocarriers, because the polymerization of pyrrole is controllable and the growth of PPy may take place on the outer or inner surface of the frame [69,71,72]. With good bioavailability and plenty of catechol groups for chemical modification, PDA may be good frames for further modification and drug loading [28,47,67]. Additionally, PDA has satisfactory photothermal properties that may cooperate with other frames or polymers [28,47,67].

## 7. Conclusions and Outlook

Compared with individual CHT or PTT per se, combinatory therapy of CHT and PTT will greatly enhance anti-tumor effects. The cooperation of both therapies may lead to synergistic effect by inhibiting (or even eradicating) solid tumors, impeding recurrence or metastasis of MDR tumors and improving prognosis. The key factor of a successful synergistic therapy is to design a well-designed, stable, bioavailable, targeting and stimuli-responsive nanocarrier with satisfactory performance. In this review, we listed more than hundred nanocarriers with various strengths and weaknesses. We classify them into two categories and analyzed their structure, preparation and effects. Furthermore, we summarized common problems that may prevent potential future in vivo studies and clinic applications.

In our opinion, nanocarriers with better stability (e.g., preventing premature leak), satisfactory safety (e.g., using biodegradable or fast-clearable materials) and precise controllability (e.g., light-controlled and sustained release) should be more popular in future. The detailed mechanism of the in vivo activities of nanocarriers and the distinctive interactions between nanocarriers and tumor tissues need to be further clarified in the future. The first FDA-approved combined nanotherapy involving PTT and CHT is also expectable.

## Figures and Tables

**Table 1:** List of Core-shell Structured Nanocarriers.

Photothermal Material	Drug(s)	Polymer	Targeting Ligand	Reference
IR780	AMD3100	PC, MCT and trilaurin	AMD3100	[3]
ICG	DOX	Carboxyl-terminated pluronic F68 and polyethyleneimine-coated perfluorocarbon	HER2 antibody	[9]
Fe_3_O_4_	DOX	PMMA and PMMA-co-DMAEMA-*co*-DVB-*co*-PEG360-*co*-AA-*co*-DS	None	[14]
IR780	SN38	IR780-mPEG and IR780-mPEG-cRGD	cRGD	[45]
RGO	Resveratrol	Folate acid-terminated PEG-phospholipid	Folate acid	[21]
Au-S nanorod	DOX, NaHCO_3_ and Salicylic acid	PVA, PLGA and TPGS	None	[23]
AuNS	Resveratrol	Resveratrol-binding lipid and CTS	None	[24]
CuS and MoS_2_	DOX	Aptamer, NH_2_-(SH)-PEG	None	[60]
PDA	DOX	PDA and PLGA	None	[26]
OA-PB NP	DOX	DSPE-PEG 2000	None	[27]
BCNP	Curcumin	TPGS	None	[48]
PPy NP	Rapamycin	DPPC, cholesterol, CHEMS and DSPE-PEG2000-Maleimide	Trastuzumab	[49]
ICG	Wedelolactone	Soybean phosphatidylcholine, Cholesterol and PEG-2000	None	[50]
NGO	DOX and Rapamycin	PEGylated lipid	None	[51]
ICG	DOX	PEG-PLGA	HER2 antibody	[61]
RGO	DOX	PVA	Lactoferrin	[64]
HMPB	DOX	Red blood cell membrane	None	[76]
Diketopyrrolopyrrole-based polymers	DOX	TPGS-CHO copolymers	None	[80]
CuS	DTX	Povidone and chitosan	Folic acid	[81]
OA-CuS-NP	DOX	Chitosan	None	[82]
ICG	DOX	PLGA and NAcHis-TPGS	None	[83]
Graphene nanosponge	DTX and Perfluorohexane	DOPE and DSPE-PEG2000-NH2	Lactoferrin	[84]
BCNP	DOX	Tween 80	None	[85]
Nanogold and Fe_3_O_4_	DOX	PSMA	None	[86]
Fe_3_O_4_	DOX	poly(N-isopropylacrylamide)-co-1-Vinyl-2-pyrrolidone	None	[87]
ICG	DOX	PPE-Chol1-HA	Hyaluronic acid	[88]
Porphyrin-phospholipid (PoP)	DOX	DOTAP and PoP	None	[89]
PDA NP	Bortezomib and DOX	pNIPAAm-co-pAAm	None	[90]
SPION (Fe_2_O_3_)	DOX	-PLGA-PEG-PLGA-urethane-SS-	Folic acid	[91]
Fe_3_O_4_	DOX	mPEG-g-PDAEAIM	None	[92]

**Table 2:** List of Frame-Coat Structured Nanocarriers.

Frame	Photothermal Material	Drug(s)	Polymer	Targeting Ligand	Reference
Monoolein	GO	DTX	CTS-PEG	GO	[11]
Si/C NP	C in Si/C NPs	DOX	PEG	None	[39]
MWNT	MWNT	DOX	CTS	Transactivator of transcription	[41]
PdNP	Gold	DOX	ZIF-8	None	[22]
GO@Gd	GO	DOX	PEG-2000	Folic acid	[79]
PDA	PDA	DOX and SN-38	PEG	None	[28]
SWNT	SWNT	DOX	PEG	None	[29]
SiNP	Polyaniline	DOX	Polyaniline	None	[30,35]
Silica	Palladium phthalocyanine	PTX	Pluroic F108	None	[31]
ZnO nanosheet	ZnO NS	DOX	PEG	Folic acid	[35]
NGO	NGO and ICG	PTX	PEG	None	[36]
MCN	MCN	DOX	PEG-PCDA	None	[37]
PDA NP	PDA NP	CP	PEG	None	[47]
Fluorinated GO	Fe_3_O_4_	DOX	FGO	None	[59]
GO	GO	Mitoxantrone	Hyaluronic acid and Pluronic	Hyaluronic acid	[63]
MWNT@PPy@Au shell	MWNT@PPy@Au shell	DOX	FA-PEG-SH	Folic acid	[94]
PPy@Mil-100(Fe)	PPy	DOX	PPy	None	[73]
PPy	PPy	DOX	Hyaluronic acid	Hyaluronic acid	[72]
PNA	PPy	DOX	Chitosan (CTS)	None	[70]
PPy@DSN	PPy	DOX	PEG	None	[71]
ZrO_2_	(PPy)	DOX	PPy	None	[69]
NGO	NGO and GNS	DOX	PDA-PEG	None	[66]
MPDA	MPDA	DOX	TPGS	None	[67]
RGO	PDA	Ara or HCPT	PDA	None	[68]
PEI and DNA	AuNR	DOX and PND	PEI and DNA	Biotin	[74]
GNP	GNP	DOX	Hairpin-like aptamer AS1411	AS1411	[75]
AuNC with tetradecanol	AuNC	DOX	Biotin-PEG-PCDA	Biotin	[77]
AuNC with tetradecanol	AuNC and ICG	DOX	PEG	Biotin	[78]
HPMO	GNR	PTX	Mesenchymal stem cells (MSCs)	MSCs per se	[93]
NGO	NGO	DOX	PEG	Octreotide	[95]
SWCNT	CNT	DOX	CS-OA and PNIPAAm	None	[96]
RGO	rGO and Bi_2_S_3_	DOX	PVP	None	[97]
Nano graphene	Nano graphene	Curcumin	Hyperbranched polyglycerol	None	[98]
SiO_2_@Au	Gold and GO	DTX	HS-PEG-NH_2_ and GO	None	[99]
Gold nanorod (AuNR)	AuNR	DOX and TMPyP4	DNA and/or AS1411	None	[100]
AuNC	AuNC	DOX	Hyaluronic acid and PEG	A54 peptide	[101]
HAuNs	HAuNs	DOX and Ce6	p(OEGMA-*co*-MEMA)	None	[102]
AuNS	AuNS	DOX	Hyaluronic acid (HA)	HA, Cationic peptide R8 and mitochondria-targeting peptide TPP-KLA	[103]
PPy@Mil-100(Fe)	PPy	DOX	PPy	None	[104]
OA-Cu_2_Te NC	Cu_2_Te	DOX	DSPE-PEG2000	None	[105]
MoO_3−*x*_ HNS	MoO_3−*x*_ HNS	Camptothecin	PEG	None	[106]
PB-NP	PB-NP	Artemisinin	Mil-100(Fe)	No	[107]
CaCO_3_	AuNR	DOX, 17-AAG Afatinib HER-2 antibody Amylase	Phospholipid, POPC Acetalated dextran	None	[108]
G (a nano gel)	CuS	DOX	G	None	[109]
CTS	Fe_3_O_4_ and ICG	DOX	CTS	None	[110]
PNIPAM, SBMA, MAA and BAC	ICG	DOX	The same as the frame	None	[111]
Fe_3_O_4_	Fe_3_O_4_	DOX	CMCTS	None	[112]
Fe_3_O_4_@mSiO_2_	Fe_3_O_4_ and CuS	DOX	PEG	Folic acid	[113]
MSN	AuNR	DOX	Hyaluronic acid	None	[114]
